# Oral Health-Related Quality of Life in Celiac Portuguese Children: a cross-sectional study

**DOI:** 10.1007/s40368-023-00842-x

**Published:** 2023-09-25

**Authors:** M. Coelho, M. Bernardo, S. Mendes

**Affiliations:** Unidade de Investigação em Ciências Orais e Biomédicas (UICOB), Faculdade de Medicina Dentária, Universidade de Lisboa, Rua Prof. Teresa Ambrósio, Cidade Universitária, 1600-277 Lisbon, Portugal

**Keywords:** Celiac Disease, Oral Health-Related Quality of Life, Oral health behaviours, Oral manifestations

## Abstract

**Purpose:**

Celiac Disease (CD) presents a wide variety of clinical signs and symptoms, including oral manifestations. This study pretended to characterize Oral Health-Related Quality of Life (OHRQoL) and reported oral manifestations in children with CD.

**Methods:**

Target-population were children with CD. An online questionnaire, applied to children’s parents, collected information about OHRQoL (using the Early Childhood Oral Health Impact Scale—ECOHIS), oral health behaviours, and history of oral manifestations. Data analysis included descriptive statistics, Mann–Whitney, Kruskall-Wallis, and Spearman correlation tests (α = 0.05).

**Results:**

The sample included 146 celiac patients, with a mean age of 10.5 years (sd = 4.1). Mean ECOHIS score was 5.2 (sd = 6.8). The most frequently reported oral manifestations were recurrent aphthous stomatitis (46.6%), dental caries (45.2%) and dental opacity (39%). About one third of the participants mentioned improvements in oral health when a gluten-free diet was introduced. Most of the reported oral manifestations had a significant association with the ECOHIS score (*p* < 0.05).

**Conclusions:**

The OHRQoL of children was good, however oral manifestations had a negative impact on OHRQoL. The most reported oral manifestations were recurrent aphthous stomatitis, dental caries, and dental opacities. Oral health professionals must be aware about the heterogeneity of the disease, to recognize oral manifestations associated and their importance in the early diagnosis to reduce complications and to an improvement in the OHRQoL of these patients.

## Introduction

Celiac Disease (CD) is an autoimmune disease, characterized by an inflammatory reaction in the small intestine, which occurs in genetically susceptible individuals with a chronic gluten intolerance (Rivera et al. 2013). Gluten is an alcohol-soluble protein found in cereals such as wheat, barley, and rye (Caio et al 2019). This disease can develop at any age and has a wide range of clinical signs and symptoms (Rivera et al. 2013; Guandalini and Assiri 2014). The diagnosis is a complex process due to the heterogeneity of the clinical manifestations (Husby et al. 2020). Gastrointestinal symptoms in CD are usually predominant, however there are many cases where extraintestinal manifestations prevail (Guandalini and Assiri 2014).

Oral health professionals can play an important role in the early diagnosis of CD, especially in cases with atypical symptoms, in which oral manifestations may be the only symptoms present (Rashid et al. 2011; Rivera et al. 2013). Oral health professionals may be valuable, not only in the diagnosis of the disease, but also by helping in the control of oral manifestations and, consequently, improving the Oral Health-Related Quality of Life (OHRQoL) of celiac patients. OHRQoL is a multidimensional concept that reflects the level of comfort that the individual experiences when performing daily activities, as well as their self-esteem and satisfaction regarding their oral health (Bennadi and Reddy 2013). In fact, oral manifestations can have a significant impact on the OHRQoL of CD patients (van Gils et al. 2017).

The study of the OHRQoL is important, particularly in children, since the oral health condition can affect their growth, self-esteem, social life and learning ability and additionally the children's oral health problems also influence the daily life of their parents/guardians (Shaghaghian et al. 2015; Freire et al. 2022). The Early Childhood Oral Health Impact Scale (ECOHIS) was developed by Pahel et al. (2007) to evaluate the impact of dental problems and treatments on the quality of life of the child and family.

Several oral manifestations have been described as associated with CD. The most frequent are enamel defects and recurrent aphthous stomatitis (Rashid et al. 2011; Rivera et al. 2013; Macho et al. 2017; Cruz et al. 2018). However, other alterations are also described in the literature, such as, delayed tooth eruption, atrophic glossitis, angular cheilitis, geographic tongue, xerostomia, burning tongue (Macho et al. 2017), oral lichen planus (Rashid et al. 2011), microdontia (Ferraz et al. 2012) and fissured tongue (Seyhan et al. 2007). A gluten-free diet can lead to an improvement in the oral manifestations of the disease (Pastore et al. 2008a, b; Ferraz et al. 2012; Macho et al. 2017; Cruz et al. 2018).

This study aims to: (1) study the OHRQoL in children with CD; (2) characterize the reported lifetime oral manifestations of CD; (3) analyze the relation between oral health behaviours and reported oral manifestations of celiac patients with OHRQoL.

## Methods

The target-population of this cross-sectional study were CD patients with pediatric age (less than 18 years-old), and Portuguese nationality. Celiac patients with additional severe systemic diseases were excluded to avoid confounding. The study was approved by the Ethics Committee of Faculdade de Medicina Dentária da Universidade de Lisboa (ref.: 202114).

Data collection was performed between March and May 2021 by an online questionnaire answered by the parents of CD patients. The questionnaire was distributed by the Portuguese Association of Celiac Patients and through social networks in CD discussion groups.

The questionnaire was developed based on a literature review (Seyhan et al. 2007; Pastore et al. 2008a, b; Ferraz et al. 2012; Rivera et al. 2013; Macho et al. 2017; Cruz et al. 2018; Rubin and Crowe 2020). In the first page the study was presented, and the objectives and procedures explained. As the questionnaire was in a digital format, the first question guaranteed the voluntary participation in the study (informed consent) and the following questions confirmed the inclusion and exclusion criteria. The questionnaire collected sociodemographic information, oral health behaviours of the CD patient, reported history of oral manifestations of the child/adolescent, and the Portuguese version of ECOHIS. Questions about the reported history of oral manifestations were simplified and explained in the questionnaire so the parents were able to understand the medical terms used. The Portuguese version of ECOHIS validated by Costa for a 12 years-old population (Costa 2013) was included in the questionnaire. The ECOHIS is used to study OHRQoL in children and consists of thirteen items, nine of which are related to the impact of oral problems on the child /adolescent (child subscale) and four items related to the impact of oral problems on the child's family (family subscale). The entire child's lifetime, from birth to the present, is to be considered. The score given to each item depends on the answer given: “Never” = 0, “Almost never” = 1, “Occasionally” = 2, “Often” = 3 and “Very often” = 4. The answer “I don't know” is considered as “missing value”. Questionnaires with more than two missing values on the child subscale and with more than one on the family subscale were excluded. The total score of ECOHIS is the sum of all individual item values, ranging from a minimum of “0” to a maximum of “52”. Lower values correspond to a lower impact of oral health on the child's and family quality of life (Pahel et al. 2007).

Before its distribution, the questionnaire was reviewed by two experts (oral health professionals with research experience), and a pre-test was performed.

Statistical analysis was made using the IBM SPSS Statistics Software (version 26). Data analyses included descriptive statistics and Mann–Whitney, Kruskall-Wallis, and Spearman correlation tests (α = 0.05).

## Results

A total of 231 responses to the questionnaire were obtained, being 85 excluded after application of inclusion and exclusion criteria. The final study sample consisted of 146 participants. The mean age of celiac patients was 10.5 years (sd = 4.1).

Table 1 shows the distribution of the sample by sociodemographic characteristics and oral health behaviours. Regarding the oral health behaviours, most of the parents (75.3%) mentioned that the children/adolescents brushed their teeth at least twice a day. More than 80% of the celiac patients visited the oral health professional once or twice a year (Table 1). Only 10.3% of the participants reported that the oral health professional made special recommendations related to the presence of CD. Of these, 53.4% advised celiac patients to give extra attention to their oral hygiene, 46.6% suggested the use of a gluten-free toothpaste, and 26.2% recommended the adoption of a less cariogenic diet.

**Table 1:** Sociodemographic characterization, oral health behaviours and parents’ oral health perception of the children/adolescents with celiac disease

	n	%
**Age (years**)		
0–5	18	12.3
6–11	66	45.2
12–17	62	42.5
**Sex**		
Female	85	58.2
Male	61	41.8
**Toothbrushing frequency**		
Less than once a day	1	0.7
Once a day	35	24.0
Twice or more a day	110	75.3
**Frequency of sugary foods/drinks intake**		
Never	1	0.7
Rarely	13	8.9
Few times/week	46	31.5
Sometimes/week	60	41.1
Many times/week	19	13.0
Everyday	7	4.8
**Frequent oral health appointment (at least 1–2 times per year)**		
Yes	124	84.9
No	22	15.1
**Oral health perception**		
Very good	39	26.7
Good	69	47.3
Reasonable	34	23.3
Bad	4	2.7
Very bad	0	0.0

Concerning the ECOHIS, most of the participants answered “Never” to the different items (Table 2). The item that revealed the greatest impact on OHRQoL was pain, with 53.4% of the parents stating that their child/adolescent had already experienced tooth/mouth pain. The mean total score of ECOHIS was 5.2 (sd = 6.8), with a minimum of “0” and a maximum of “44”. The child subscale mean score was 3.4 (sd = 4.5), with “0” as the minimum value and “28” as the maximum. Regarding the family subscale, 43.4% of parents admitted that their child's dental treatment had an impact on the family budget. The mean score of the family subscale was 2.0 (sd = 2.7), with a minimum of “0” and a maximum of “16” (Table 2).

**Table 2:** ECOHIS: items frequencies and mean scores

	Item	Never % (n)	Hardly never % (n)	Occasionally %(n)	Often % (n)	Very often % (n)	Mean (sd)
Child subscale items	1. How often has your child had pain in the teeth, mouth, or jaws?	46.6 (68)	39 (57)	12.3 (18)	0.7 (1)	1.4 (2)	0.7 (0.8)
	2. How often has your child had difficulty drinking hot or cold beverages because of dental problems or dental treatments?	61.4 (89)	30.3 (44)	6.2 (9)	0.7 (1)	1.4 (2)	0.5 (0.8)
	3. How often has your child had difficulty eating some foods because of dental problems or dental treatments?	66.9 (97)	25.5 (37)	5.5 (8)	0.7 (1)	1.4 (2)	0.4 (0.8)
	4. How often has your child had difficulty pronouncing any words because of dental problems or dental treatments?	82.9 (121)	13 (19)	3.4 (5)	0 (0)	0.7 (1)	0.2 (0.6)
	5. How often has your child missed preschool, daycare or school because of dental problems or dental treatments?	81.5 (119)	14.4 (21)	3.4 (5)	0.7 (1)	0 (0)	0.2 (0.5)
	6. How often has your child had trouble sleeping because of dental problems or dental treatments?	76.7 (112)	19.9 (29)	3.4 (5)	0 (0)	0 (0)	0.3 (0.5)
	7. How often has your child been irritable or frustrated because of dental problems or dental treatments?	70.5 (103)	19.9 (29)	5.5 (8)	2.7 (4)	1.4 (2)	0.4 (0.8)
	8. How often has your child avoided smiling or laughing because of dental problems or dental treatments?	82.1 (119)	9.7 (14)	6.2 (9)	2.1 (3)	0 (0)	0.3 (0.7)
	9. How often has your child avoided talking because of dental problems or dental treatments?	83.4 (121)	12.4 (18)	2.8 (4)	0.7 (1)	0.7 (1)	0.2 (0.6)
Family subscale items	10. How often have you or another family member been upset because of your child's dental problems or treatments?	64.8 (94)	24.8 (36)	6.9 (10)	2.1 (3)	1.4 (2)	0.5 (0.8)
	11. How often have you or another family member felt guilty because of your child's dental problems or treatments?	76.6 (111)	14.5 (21)	6.9 (10)	0.7 (1)	1.4 (2)	0.4 (0.8)
	12. How often have you or another family member taken time off from work because of your child's dental problems or treatments?	76.6 (111)	19.4 (28)	2.8 (4)	0.7 (1)	0.7 (1)	0.3 (0.7)
	13. How often has your child had dental problems or dental treatments that had a financial impact on your family?	56.6 (82)	26.2 (38)	6.9 (10)	4.8 (7)	5.5 (8)	0.8 (1.1)
Total ECOHIS score		–	–	–	–	–	**5.2 (6.8)**
Child subscale score		–	–	–	–	–	**3.4 (4.5)**
Family subscale score		–	–	–	–	–	**2.0 (2.7)**

**Fig. 1:**
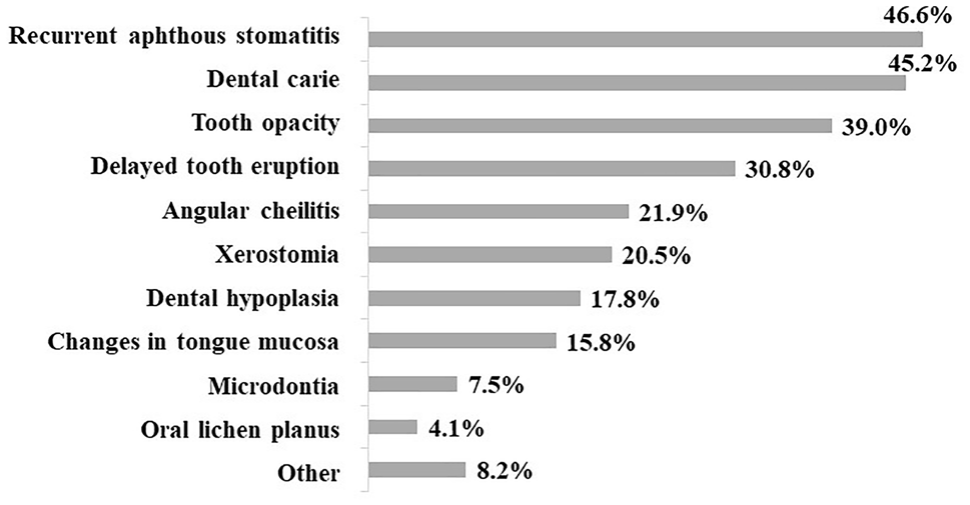
Lifetime oral manifestations reported by parents of pediatric celiac patients

The percentage of children/adolescents with a lifetime history of at least one reported oral manifestation of CD was 87.0%. The mean number of reported oral manifestations per individual was 2.6 (sd = 2.0), with a minimum value of “0” and a maximum of “10”. The most frequently reported oral manifestations were recurrent aphthous stomatitis (46.6%) and dental caries (45.2%) (Fig. 1). Close to one third of the participants (32.4%) claimed that there were oral health improvements when the gluten-free diet was introduced, with the main improvement reported being a decrease in recurrent aphthous stomatitis (50.6%).

No associations were found between oral health behaviours and OHRQoL. There was a significant and direct association between oral health perception and OHRQoL (*p* < 0.001) (Table 3).

**Table 3:** Relation between sociodemographic and behavioural factors and Oral Health-Related Quality of Life (ECOHIS)

	Child subscale		Family subscale		Total ECOHIS	
	Mean (sd)	*p*	Mean (sd)	*p*	Mean (sd)	*p*
**Age (years)**						
0–5	1.8 (2.5)	0.3*	1.3 (2.1)	0.3*	3.1 (4.2)	0.4*
6–11	3.1 (3.8)		2.2 (2.7)		5.2 (5.8)	
12–17	4.1 (5.5)		1.8 (2.9)		5.9 (8.3)	
**Sex**						
Female	3.2 (3.9)	0.8**	1.8 (2.4)	0.9**	4.9 (5.6)	0.9**
Male	3.6 (5.4)		2.1 (3.1)		5.7 (8.2)	
**Toothbrush frequency**						
One time/day or less	3.5 (3.5)	0.2**	1.9 (2.0)	0.4**	5.4 (5.1)	0.4**
Twice or more/day	3.3 (4.9)		2 (2.9)		5.2 (7.3)	
**Frequency of sugary foods/drinks intake**						
Never/rarely	3.1 (3.3)	0.6*	1.0 (1.3)	0.8*	4.1 (4.3)	0.6*
A few times/week	3.5 (4.4)		2.3 (3.0)		5.9 (7.1)	
Sometimes/week	3.8 (5.3)		2.0 (2.7)		5.7 (7.7)	
Many times/week	2.2 (3.1)		1.8 (2.8)		4.1 (4.9)	
Everyday	2.1 (3.7)		1.7 (2.6)		3.9 (6.3)	
**Frequent oral health appointment (at least one time per year)**						
Yes	3.5 (4.7)	0.4**	2.1 (2.8)	0.08**	5.6 (7.1)	0.1**
No	2.4 (3.2)		1.1 (1.8)		3.5 (4.8)	
**Oral health perception**						
Very good	1.5 (3.8)	< 0.001*	0.9 (2.1)	< 0.001*	2.5 (5.7)	< 0.001*
Good	3.0 (3.6)		1.7 (2.4)		4.7 (5.4)	
Reasonable	6.1 (5.8)		3.4 (3.3)		9.3 (8.6)	
Bad	4.3 (4.9)		4.3 (3.4)		8.5 (7.7)	

*p* values in bold are statistically significant

*Kruskal–Wallis, **Mann–Whitney

There was also a significant association between the total ECOHIS score and almost all reported oral manifestations (*p* < 0.05) (Table 4), confirming that the presence of oral manifestations can have a highly negative impact on the OHRQoL of the children and their families. Children/adolescents who had at least one reported oral manifestation had a worse OHRQoL when compared to those who reported no oral manifestations (*p* < 0.001).

**Table 4:** Relation between reported oral manifestations and Oral Health-Related Quality of Life (ECOHIS)

	Child subscale		Family subscale		Total ECOHIS	
	Mean (sd)	*p*	Mean (sd)	*p*	Mean (sd)	*p*
Dental hypoplasia						
Yes	4.1 (4.7)	0.1**	2.8 (2.8)	0.02**	6.7 (6.9)	0.05**
No	3.2 (4.5)		1.8 (2.7)		5 (6.8)	
Dental opacity						
Yes	4.7 (5.6)	0.005**	3.0 (3.1)	< 0.001**	7.6 (8.4)	< 0.001**
No	2.5 (3.5)		1.3 (2.2)		3.7 (5.1)	
Dental caries						
Yes	4.5 (4.3)	< 0.001**	2.5 (2.6)	< 0.001**	7 (6.3)	< 0.001**
No	2.4 (4.6)		1.5 (2.7)		3.8 (6.9)	
Delay in teeth eruption						
Yes	4.3 (4.9)	0.06**	2.8 (2.9)	0.003**	7.1 (7.4)	0.010**
No	2.9 (4.3)		1.6 (2.6)		4.4 (6.4)	
Microdontia						
Yes	5.9 (4)	0.02**	3.8 (2.4)	0.005**	9.7 (5.8)	0.010**
No	3.1 (4.5)		1.8 (2.7)		4.6 (6.8)	
Recurrent aphthous stomatitis						
Yes	4.3 (5.4)	0.04**	2.5 (3.0)	0.006**	6.8 (7.9)	0.006**
No	2.5 (3.5)		1.5 (2.4)		3.9 (5.4)	
Changes in tongue mucosa						
Yes	6.8 (6.9)	< 0.001**	3.7 (3.9)	0.007**	10.5 (10.7)	0.001**
No	2.7 (3.6)		1.6 (2.3)		4.3 (5.3)	
Angular cheilitis						
Yes	5.6 (6.5)	0.012**	3.1 (3.7)	0.012**	8.7 (9.9)	0.005**
No	2.7 (3.6)		1.6 (2.3)		4.3 (5.3)	
Oral lichen planus						
Yes	3.7 (3.8)	0.5**	2.7 (3.4)	0.6**	6.3 (6.9)	0.5**
No	3.3 (4.6)		1.9 (2.7)		5.2 (6.8)	
Xerostomia						
Yes	6.5 (6.1)	< 0.001**	3.3 (3.7)	0.002**	9.8 (9.6)	< 0.001**
No	2.5 (3.7)		1.6 (2.3)		4.1 (5.3)	
At least one oral health manifestation						
Yes	3.8 (4.7)	< 0.001**	2.2 (2.8)	< 0.001**	5.9 (7.0)	< 0.001**
No	0.5 (2.1)		0.2 (0.9)		0.7 (3.0)	

*p* values in bold are statistically significant

**Mann–Whitney

When analyzing the correlation between the number of oral manifestations and the total score of ECOHIS, a significant and direct relationship was found (*p* < 0.001; ρ = 0.5), revealing that the greater the number of reported oral manifestations, the worse the OHRQoL of the celiac patient.

## Discussion

Celiac Disease is an underdiagnosed pathology, partially due to the wide variety and non-specificity of clinical signs and symptoms (Pastore et al. 2008a, b; Lindfors et al. 2019). Oral manifestations can be important for the diagnosis of CD, especially in atypical clinical cases (Jajam et al. 2017). In these atypical cases, oral manifestations may be the only signs and symptoms present, and the oral health professional may be important in the diagnosis, control, and counselling of these patients. When there is suspicion of CD, the dentist or dental hygienist should advise the patient to seek a gastroenterologist, to screen for this autoimmune disease (Costacurta et al. 2010; Macho et al.^2017^). Additionally, in patients already diagnosed, the oral health professional should be aware of the components of the products they use in their clinical practice to ensure that they do not contain gluten. Also, more specific recommendations must be made to these patients, to indicate safe oral hygiene products and to make frequent control appointments to reduce possible oral manifestations and their consequences. The oral health professional must reinforce the importance of a gluten-free diet, which is to date, the only effective form of control for this disease.

Oral health behaviours were generally well-implemented in celiac children, with the majority performing twice a day toothbrushing, having low frequency of cariogenic foods and drinks intake, and frequent appointments to the oral health professional. Shteyer et al. (2013) determined that 66.7% of celiac children brushed their teeth twice a day, compared to 60% of the healthy children. Toothbrushing with a fluoride paste is an important recommendation for all patients but is especially important for celiac patients who have frequently enamel defects, which increase the risk of developing dental caries.

The assessment of OHRQoL to measure the impact of oral pathologies reflects a more focused and patient-centered follow-up and treatment. Although ECOHIS values in the present study can be considered low, revealing a good OHRQoL of the celiac children, almost all the reported oral manifestations showed a relationship with a worse OHRQoL. The relationship between OHRQoL and CD was suggested by van Gils et al. (2017) who verified a clear impact of CD on Health-Related Quality of Life, when compared to a group of healthy individuals.

In the present study, several oral manifestations were reported, and the oral health professional should be aware of those manifestations. Oral health manifestations are described in a high percentage of celiac patients (Campisi et al. 2008; Nieri et al. 2017; Villemur Moreau et al. 2021). Recurrent aphthous stomatitis is one of the oral manifestations most frequently described in CD, and its etiology is thought to be related to anemia and hematinic deficiencies (Jajam et al. 2017). According to the literature, there is a decrease or even total regression of recurrent aphthous stomatitis with the introduction of a gluten-free diet (Bucci et al. 2006; Campisi et al. 2008; Pastore et al. 2008a, b), as reported by the parents of celiac children in the present study.

Dental manifestations were also among the most reported oral manifestations in the studied population. The prevalence of these manifestations in the literature ranges between 10 and 96% (Pastore et al. 2008a, b; Rashid et al. 2011; Ferraz et al. 2012; Macho et al. 2017). A meta-analysis (Nieri et al. 2017) described that a study with 1490 celiac children and 2318 healthy children, demonstrated that 46% of children with CD were found to have at least one affected tooth with enamel defects, compared to 14% of children in the control group. The etiology of dental enamel defects in celiac patients is not yet clarified (Bramanti et al. 2014). These dental alterations may be associated with several systemic diseases and not just CD, but the defects present in celiac patients are highly specific, being characterized by dotted surfaces, furrows, and sometimes complete loss of enamel (Macho et al. 2017). de Carvalho et al. (2015). De Carvalho et al. (2015) analyzed the chemical composition of the enamel of primary teeth in patients with CD and found that the calcium/phosphorus ratio is significantly lower in these individuals. These defects can result from hypocalcemia, genetic susceptibility, or an autoimmune reaction in the enamel organ during tooth formation, before the 7th year of age (Macho et al. 2017; Cruz et al. 2018).

The association between dental caries and CD is still controversial, with some studies showing a higher caries experience in celiac patients (Costacurta et al. 2010) and others the opposite (de Carvalho et al. 2015). Some authors refer that dental caries are less prevalent in these patients due to strict diet control. Other authors justify the association of CD with dental caries due to the fragility of the hypoplastic enamel and changes in saliva composition or reduced salivary flow (Macho et al. 2017). Despite contradictory data and authors opinions, oral health professionals should consider incorporating preventive oral health measures in these patients, which may include topical fluoride and sealants, as well as early treatment of caries lesions and fractures of the hypoplastic enamel (Macho et al. 2017).

The impact of oral manifestations on the OHRQoL of celiac children/adolescents was lower than the one reported by Mansoori et al. (2019) in Indian children aged 0 to 6 years (ECOHIS score = 7.0), but higher than the value obtained by Chaffee et al. (2017) in Brazilian children aged 2 and 3 years (ECOHIS score = 2.0).

The negative impact of oral manifestations of CD on OHRQoL can be explained in several ways. Recurrent aphthous stomatitis can affect diet, speech, as well as toothbrushing and, in addition, it can cause emotional instability due to pain and discomfort (Macho et al. 2017). Patients with atrophic glossitis also report difficulty in chewing, swallowing, and talking (Bucci et al. 2006; Macho et al. 2017). On the other hand, enamel defects cause discomfort to patients and put them at risk for oral pathologies such as dental caries (Cervino et al. 2018). Children with caries lesions are more likely to have pain and chewing difficulties, which can lead to greater concern with oral health (Piovesan et al. 2011). In this study, as in the studies of Piovesan et al. (2011), Chaffee et al. (2017), and Freire et al. (2022) caries experience was associated with a worse OHRQoL.

The associations found between the reported oral manifestations and the OHRQoL of CD patients highlight the importance of a correct diagnosis of the pathology and, consequently, of its control with a gluten-free diet.

This cross-sectional study included a non-probabilistic sample and analyzed the perceived oral health of celiac children by their parents. The DC is described as an under diagnosed disease with a prevalence of about 1% of the general population. Considering the Portuguese population under 18 years-old (1,800,000) (PORDATA 2021) and a prevalence of DC 0.7% in the Portuguese population (Antunes et al. 2006) the present study included about 0.8% of the celiac population in Portugal (PORDATA 2021). Additionally, the data were collected by an online questionnaire and this methodology have some advantages, but also several caveats. The latter ones are related to the sample that can include the possibility of self-selection, non-response, unknown participation rates, and under-coverage of the target population, determined, amongst others, by access to the internet (Nayak and Narayan 2019). Despite its limitations, this study may provide a valuable insight of the OHRQoL in celiac children and of oral manifestations associated with CD, particularly in the Portuguese population, where these studies are scarce. It also highlights the importance of the oral health professionals to be aware of the heterogenous presentation of the disease, and their role in early diagnosis, leading to a reduction of complications and a consequent improvement in the OHRQoL of CD patients.

## Conclusions

Considering the limitations of the present study the following conclusions can be made:

- Reported lifetime oral manifestations were frequent in children with CD, being the most frequently reported recurrent aphthous stomatitis and dental caries.
- The OHRQoL of children was good, however, most of the reported oral manifestations had a negative impact on OHRQoL.
- These patients felt an improvement in their oral health after the introduction of the gluten-free diet, especially concerning the recurrent aphthous stomatitis.

## Data Availability

Authors declare data transparency and if necessary, availability of data and material.
